# Probiotics relieve perioperative postoperative cognitive dysfunction induced by cardiopulmonary bypass through the kynurenine metabolic pathway

**DOI:** 10.1038/s41598-024-59275-1

**Published:** 2024-06-04

**Authors:** Xiaodong Zhang, Yanzhang Yang, Xinyi Ma, Huijuan Cao, Yingjie Sun

**Affiliations:** 1 Department of Anesthesiology, Xiangyang Central Hospital, Affiliated Hospital of Hubei University of Arts and Science, Xiangyang, 441000 Hubei China; 2 Postgraduate Training Base, The General Hospital of Northern Theater Command, Jinzhou Medical University, Jinzhou, 121013 Liaoning China; 3 Department of Anesthesiology, Chifeng Municipal Hospital, Chifeng, 024000 Inner Mongolia China; 4 Postgraduate Training Base, The General Hospital of Northern Theater Command, Dalian Medical University, Dalian, 116051 Liaoning China; 5 Department of Anesthesiology, General Hospital of Northern Theater Command, Shenyang, 110016 Liaoning China

**Keywords:** Probiotics, Postoperative cognitive dysfunction, Cardiopulmonary bypass, Kynurenine metabolic pathway, Microbiology, Molecular medicine

## Abstract

Postoperative cognitive dysfunction (POCD) has become the popular critical post-operative consequences, especially cardiopulmonary bypass surgery, leading to an increased risk of mortality. However, no therapeutic effect about POCD. Probiotics are beneficial bacteria living in the gut and help to reduce the risk of POCD. However, the detailed mechanism is still not entirely known. Therefore, our research aims to uncover the effect and mechanism of probiotics in relieving POCD and to figure out the possible relationship between kynurenine metabolic pathway. 36 rats were grouped into three groups: sham operated group (S group, n = 12), Cardiopulmonary bypass group (CPB group, n = 12), and probiotics+CPB (P group, n = 12). After CPB model preparation, water maze test and Garcia score scale was performed to identify the neurological function. Immunofluorescence and Hematoxylin and eosin staining has been used for hippocampal neurons detection. Brain injury related proteins, oxidative stress factors, and inflammatory factors were detected using enzyme-linked immunosorbent assays (ELISA). Neuronal apoptosis was detected by TdT-mediated dUTP nick end-labeling (TUNEL) staining and western blot. High-performance liquid chromatography/mass spectrometry (HPLC/MS) was performed to detect the key factors of the kynurenine metabolic pathway. Our results demonstrated that probiotics improved neurological function of post-CPB rats. The administration of probiotics ameliorated memory and learning in spatial terms CPB rats (P < 0.05). Hematoxylin and eosin (H&E) staining data, S‐100β and neuron-specific enolase (NSE) data convinced that probiotics agonists reduced brain damage in CPB rats (P < 0.05). Moreover, probiotics regulated inflammatory factors, meanwhile attenuated hippocampal neuronal apoptosis. Probiotics alleviated POCD in rats with CPB through regulation of kynurenine metabolic signaling pathway.

## Introduction

Cardiopulmonary bypass (CPB) is widely used in cardiac surgery that diverts the patient's blood into an external circuit replacing the function of heart^1^. Despite development for decades, CPB still lead to neurologic complications, such as stroke^2^, seizures^3^, delirium^4^, phrenic nerve palsy^5^, and perioperative neurocognitive disorders (PND)^6^. PND involves in preoperatively or postoperatively cognitive impairment or cognitive changes. PND contains the preoperatively diagnosed cognitive impairment, postoperative delirium (POD), cognitive decline, postoperative cognitive dysfunction (POCD) and neurocognitive disorders (NCD)^7^. CPB has independently contributed to POCD with incidence up to 60%^8^. Moreover, CPB may cause the inflammation-associated hippocampal damage and lead to POCD^9^. Interestingly, POCD is effectively relieved after the treatment of systemic inflammatory response syndrome (SIRS)^10,11^. However, the detailed pathogenesis and complexity of POCD is still not fully discovered.

SIRS has been implicated in blood–brain barrier leakage, cerebral edema, and inflammation^12,13^, and has become a regulator in the progress of POCD^14,15^. Recent studies have attempted to confirm the involvement of SIRS in POCD by administering various doses and types of corticosteroids during the perioperative period to reduce inflammation and lower the incidence of POCD^14,15^. In the inflammatory therapy, probiotics regulate the host’s gut microbiota, reduce the inflammatory response, and thus play a role in preventing POCD. In addition, probiotics could also enhance gut barrier function, prevent harmful substances from entering the bloodstream, and protect the brain from damage^16^. Several researchers reported that oral probiotics can reduce the incidence of POCD^16–18^. A study on cardiac surgery patients found that oral probiotics significantly reduced the incidence of POCD and improved inflammatory markers^19^. In addition, another study found that oral probiotics improved cognitive function and reduced the risk of POCD in elderly patients^20^. Although current research indicates that probiotics may have potential in preventing POCD, more researches are needed to determine their molecule mechanism, and to provide more conclusive evidence for probiotics’ promotion and application. The kynurenine pathway (KP) was involved in regulating the cognitive function^21–23^. Under normal physiological conditions, tryptophan (TRP) is metabolized by tryptophan-2,3-dioxygenase (TDO), which helps to maintain the KP in a dynamic equilibrium. However, during inflammatory states, the increasing inflammatory factors stimulate the activity of indoleamine-2,3-dioxygenase (IDO), which promotes degradation of TRP. As a result, IDO becomes the primary rate-limiting enzyme in the KP for the breakdown of TRP. An imbalance of kynurenic acid (KYNA) and quinolinic acid (QUIN) can affect central nervous system and regulate cognitive function^24,25^. Those reports suggest that the abnormal metabolism of the KP pathway due to systemic inflammation following CPB may contribute to the development of POCD.

According to our preliminary research, pre-treatment with probiotics can improve intestinal barrier function to a certain extent in rats after CPB^26^. A possible explanation for this effect is that CPB inhibits CPB-induced inflammatory responses, improves local intestinal immune function, and promotes the levels of tight junction proteins^26^. Therefore, our research aims to investigate the function of probiotics on the KP related molecules, inflammatory factors, and cognitive function in rats after CPB by establishing CPB model. We explored whether probiotics improve cognitive function after CPB by regulating peripheral blood TRP and KYNA, and seeks to identify new interventions targeting the gut microbiota to improve cognitive function after CPB, providing experimental evidence for its clinical application.

## Materials and methods

### Experimental animals and groupings

Age matched 36 Sprague–Dawley male (n = 18) and female (n = 18) rats (6–8 weeks old; 300–350 g for males and 250–300 g for females) were used in this experiment. All the rats were procured from the animal center of General Hospital of Northern Theater Command. The protocols of animals were approved by the Ethics Committee of Experimental Animal Welfare (No. 2020079). The study was carried out obeying the guidelines of ARRIVE. Rats were categorized into three distinct groups (n = 12 each group): Sham operation (Sham group), CPB surgery (CPB group), probiotics (P group). One week before surgery, rats of P group were orally administered 2 ml of Jin Shuang qi suspension (a probiotic preparation containing live bacteria of *Bifidobacterium*, *Streptococcus thermophilus*, and *Lactobacillus*) with a concentration of 1 × 10^8^/ml per day. Rats of the S and C groups were orally administered 2 ml of saline. Anesthesia is initiated via an intraperitoneal injection of pentobarbital sodium, administered at a dosage of 50 mg/kg. After anesthesia, all rats underwent mechanical ventilation and intravenous puncture. Rats of S group did not undergo CPB surgery, while rats of the C and P groups underwent CPB for 1 h. Finally, euthanasia of the rats was performed through anaesthesia with isoflurane (4–5%; oxygen at 2 L per minute) followed by decapitation by guillotine. The bilateral hippocampus was immediately collected for further studies. No animals were sacrificed during the current study.

### CPB model preparation procedure

In accordance with previous study, CPB surgery was performed^27^. The preparation involves the following steps: anesthesia is initiated via an intraperitoneal injection of pentobarbital sodium, administered at a dosage of 50 mg/kg. Endotracheal intubation is performed using a 16-gauge catheter. A midline incision is made in the chest and cannulation is done in the right jugular vein and carotid artery using a 22-gauge needle and a 24-gauge needle respectively. Heparinization is performed by administering heparin (300 IU/kg) through the jugular vein. The rats are then placed on a bypass circuit consisting of a roller pump, an oxygenator, and a heat exchanger, draining blood from the inferior vena cava and returning it to the ascending aorta. Throughout the CPB period, detection of mean arterial pressure (MAP), central venous pressure (CVP), and blood gases is carried out. After 60 min of CPB, the rats were weaned off bypass and the chest were closed.

### Water maze test

The experimental procedure involves setting up a large circular pool with a hidden platform just below the water’s surface. Rats are allowed to habituate to the water before the acquisition phase begins, where they are trained to locate the hidden platform using spatial cues. During the probe trial, the platform is removed, and rats’ behavior was recorded to assess their memory. Data analysis includes recording and analyzing swim paths, escape latencies, and time spent in each quadrant of the pool during the acquisition and probe trials.

### Neurological function scores

Following one, three, and seven days of preparing the CPB model, Garcia score scale was employed to assess the neurological functions of rats across various groups^28,29^.

### Hematoxylin and eosin (H&E) staining

Following with collection, hippocampus tissues were immobilized in a 4% paraformaldehyde and embedded. 5 µm sections were sliced. To prepare the sections for observation, they were dewaxed using xylene, and stained with hematoxylin (Solarbio, Beijing, China), followed by rinsing with tap water. Slices were then subjected to ethanol hydrochloride treatment for 30 s, and stained with eosin. After transparentization and sealing, hippocampal CA1 region were observed under Olympus BX51 optical microscope (Olympus Company, Japan) at a magnification of ×400.

### TdT-mediated dUTP nick end-labeling (TUNEL) protocol

Protocol strictly followed with the Roche protocol (Roche diagnostics GmbH, Mannheim, Germany). The data were observed using an excitation wavelength ranging from 520 to 560 nm (maximum 540; green) and 570–620 nm (maximum 580 nm; red).

### Enzyme-linked immunosorbent assays (ELISA)

Blood was centrifuged to isolate the supernatant. interleukin (IL)-1β, IL-6, tumor necrosis factor alpha (TNF-α), and Interferon (IFN) -γ was detected using the corresponding kits, following the manufacturer's instructions strictly. The soluble protein-100β (S-100β) and NSE levels were detected by S-100β ELISA kit (R&D systems, Minneapolis, MN, USA).

### High-performance liquid chromatography-tandem mass spectrometry (HPLC–MS/MS)

The quantitative analysis of TRP and kynurenine (KYN) was performed using HPLC–MS/MS. Both analytes were quantified using the peak area measurement method with a standard curve and the data were acquired and processed using Agilent Mass Hunter workstation. To prepare the samples, 50 μL of rat plasma was mixed with 50 μL of ultra-pure water and then 200 μL of acetonitrile was added for protein precipitation. For sample analysis, 20 μL of the supernatant was injected into the chromatographic system. The chromatographic conditions were as follows: a Waters HSS T3 column (100 mm × 2.1 mm, 1.8 μm), a mobile phase consisting of acetonitrile and 10 mmol/L ammonium acetate solution (containing 0.1% formic acid) in 0.5 mL/min, and an injection volume of 10 μL. Prior to analysis, 200 μL of each sample was mixed with 20 μL of 10% HClO_4_ precipitant, vortexed, centrifuged to obtain the supernatant for injection.

### Statistical analysis

Data were processed using SPSS19.0. Normally distributed metric data were expressed as mean ± standard deviation (X ± SD). All experimental results of the two-subgroup design were analyzed through independent student *t*-test. The results of the multi-group design were determined by one-way ANOVA and post-hoc Tukey test of multiple comparisons. P < 0.05 was considered statistically significant.

### Ethics approval and consent to participate

This study and included experimental procedures were approved by the institutional animal care and use committee of General Hospital of Northern Theater Command. All animal housing and experiments conducted in strict accordance with the institutional guidelines for care and use of laboratory animals. The experimental protocol/s was approved by General Hospital of Northern Theater Command ethical committee on October 20, 2020.

## Results

### CPB rat model performs the neurological deficit

**Figure 1:**
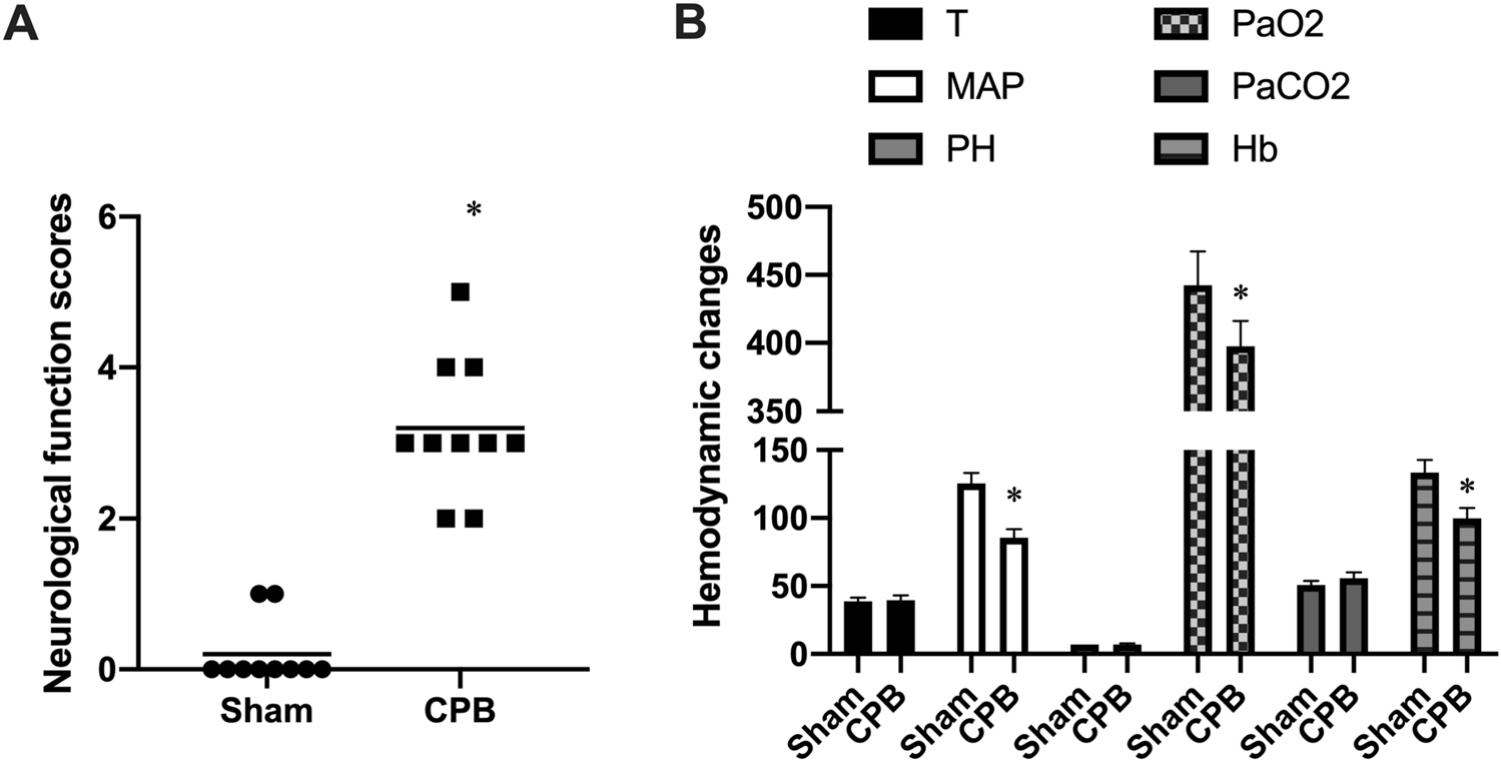
CPB rats were prepared successfully. The CPB model was used to study rats, and their neurological function scores and hemodynamic parameters were evaluated after the procedure. (**A**) The neurological function scores of rats in the sham and CPB groups. (**B**) Hemodynamic parameters after CPB surgery. *P < 0.05.

All rats regained consciousness within 90 min after surgery. As illustrated in Fig. 1A, rats in the Sham group exhibited no discernible neurological impairments, while those in CPB group displayed varying degrees of neurological abnormalities. The neurological deficit scores, as shown in Fig. 1A, significantly increased after CPB (P < 0.05) compared to sham group. The CPB model rats maintained stable data for rectal temperature, pH, and arterial blood partial pressure of carbon dioxide (PaCO_2_), but there was reduction in partial pressure of oxygen (PaO_2_) and hemoglobin levels, as depicted in Fig. 1B. Our findings imply that the CPB rat model was effective set up, and CPB surgery unequivocally resulted in injury to the rats’ neurological system.

### Probiotics alleviate the neurological dysfunction in CPB rats

**Figure 2:**
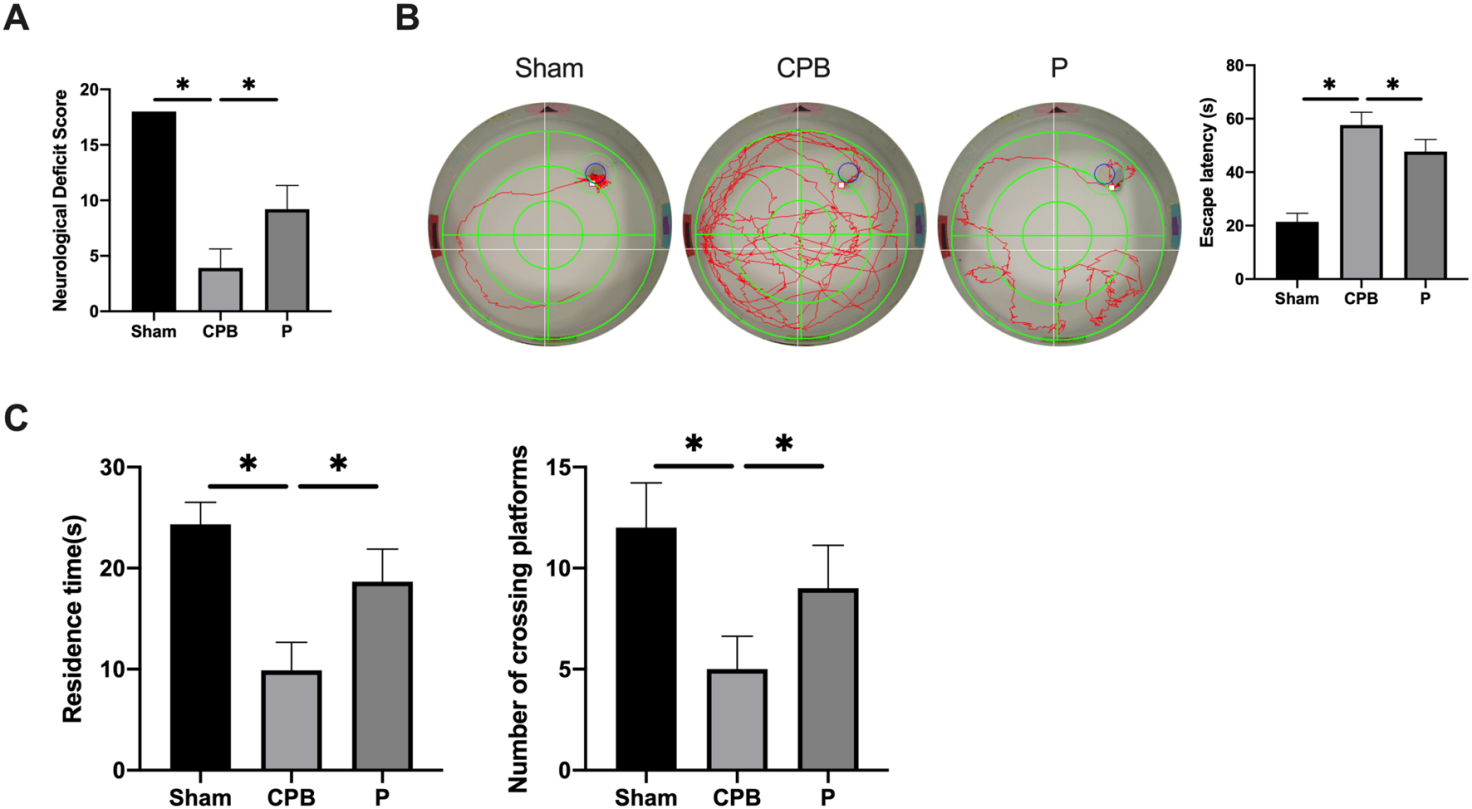
The administration of probiotics has been shown to alleviate neurological dysfunctions in rats undergoing CPB. To evaluate postoperative cognitive dysfunction, we conducted water maze tests and measured neurological function scores. The results were presented as follows: (**A**) neurological function score, (**B**) hidden platform training test, and (**C**) space exploration experiment. *P < 0.05. *CPB* cardiopulmonary bypass.

The administration of probiotics resulted in an enhancement of the neurological score within the probiotics group as compared to the CPB group (Fig. 2A). To evaluate cognitive function, we conducted the water maze test (Fig. 2B, C). Our data indicated that rats of CPB group exhibited prolonged latency in locating the platform compared to that in Sham group (P < 0.05). Conversely, the H group demonstrated a significantly shorter latency in finding the platform compared to the CPB group (P < 0.05). In the spatial exploration experiment, the CPB group displayed a notable reduction in the duration of staying in the original station quadrant and the number of times the original station was crossed in the target quadrant compared to the Sham group (P < 0.05). In contrast, the H group showed a significant increase in both the duration and frequency of staying in the original station quadrant compared to the CPB group (P < 0.05). The observed conclusions convinced that probiotics have the potential to alleviate neurological dysfunction.

### Probiotics reduced the brain tissue injury in CPB rats

**Figure 3:**
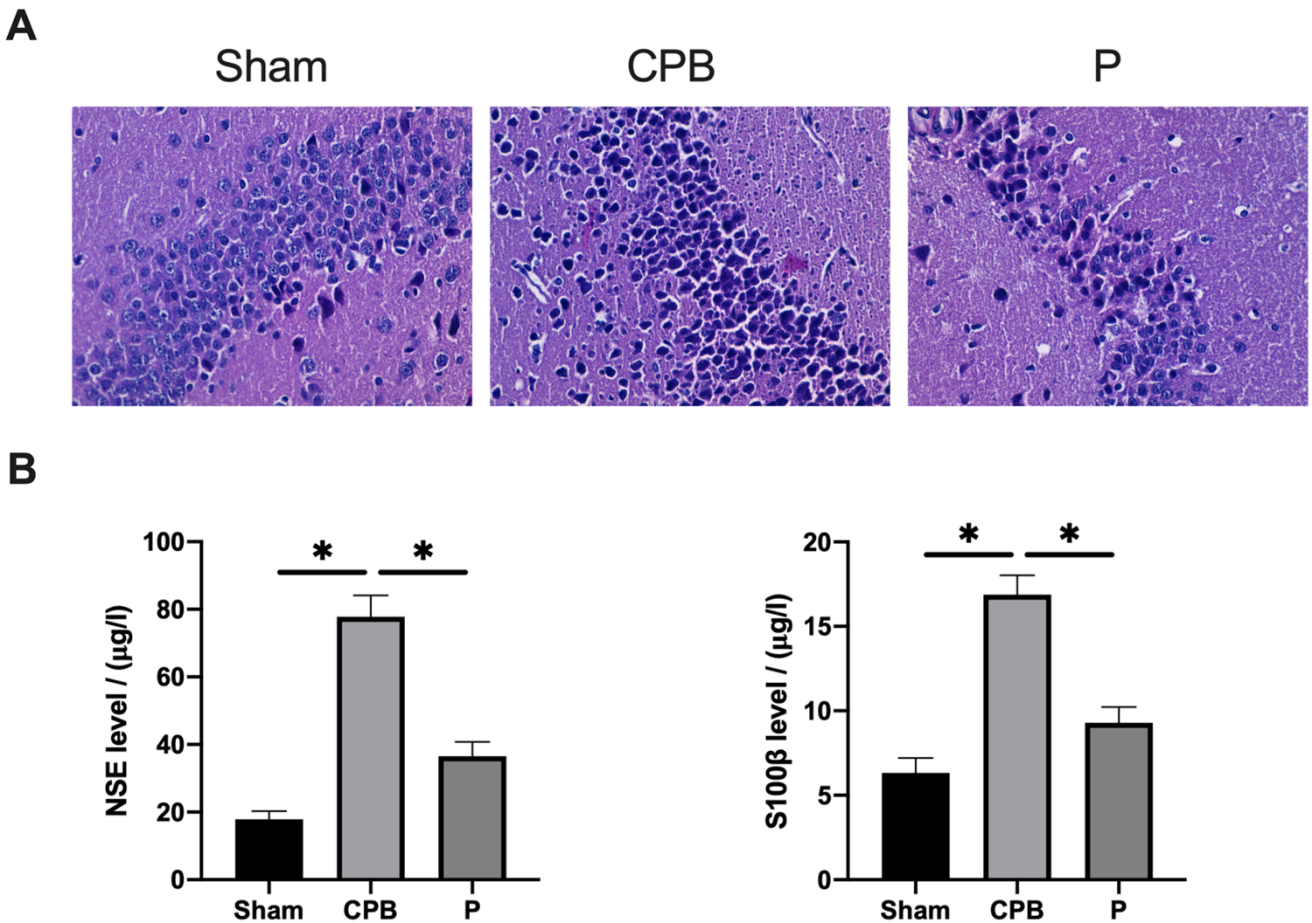
The effects of probiotics on brain dysfunction in CPB rats were assessed using H&E staining and ELISA. (**A**) H&E results (scale bar = 50 μm). (**B**) ELISA data, *P < 0.05 indicating significance.

To evaluate the benificial impact of probiotics on brain tissue injury resulting from CPB, we utilized H&E staining to analyze hippocampal neurons (Fig. 3A). In the Sham group, hippocampal neurons exhibited a well-organized and tightly packed arrangement, characterized by clear boundaries and intact cell bands. Cells displayed normal structures, with distinct cytoplasmic and nuclear staining, indicating the absence of apparent lesions. In contrast, the hippocampus of CPB group rats showed significant damage, with disorganized cells, widened intercellular spaces, and increased astrocyte and vascular proliferation. Nerve cells appeared disarrayed, with nucleus dissolution, noticeable neuronal cell and cone cell death, resulting in a considerable reduction in cell count in the hippocampus. However, in the P group, the arrangement of hippocampal cells was more regular than in the CPB group, and the number of degenerative/necrotic nerve cells was significantly lower. Brain tissue staining demonstrated the less damage after probiotics therapy as neatly arranged cells and intact cell bands. These observations imply that CPB may induce severe damage to rat hippocampus, while probiotics may exert a protective effect against such damage.

To delve further into brain damage, we employed ELISA to investigate alterations in the expression of indicators for brain damage (Fig. 3B). Increased serum levels of NSE and S-100β were noted in the CPB groups compared to the Sham group (P < 0.05). However, concentrations of NSE and S-100β in the P group were notably lower than those in the CPB group (P < 0.05). These results suggest that the administration of probiotics has the capability to alleviate brain damage in CPB rats.

### Probiotics decrease the inflammation and oxidative stress in CPB rats

**Figure 4:**
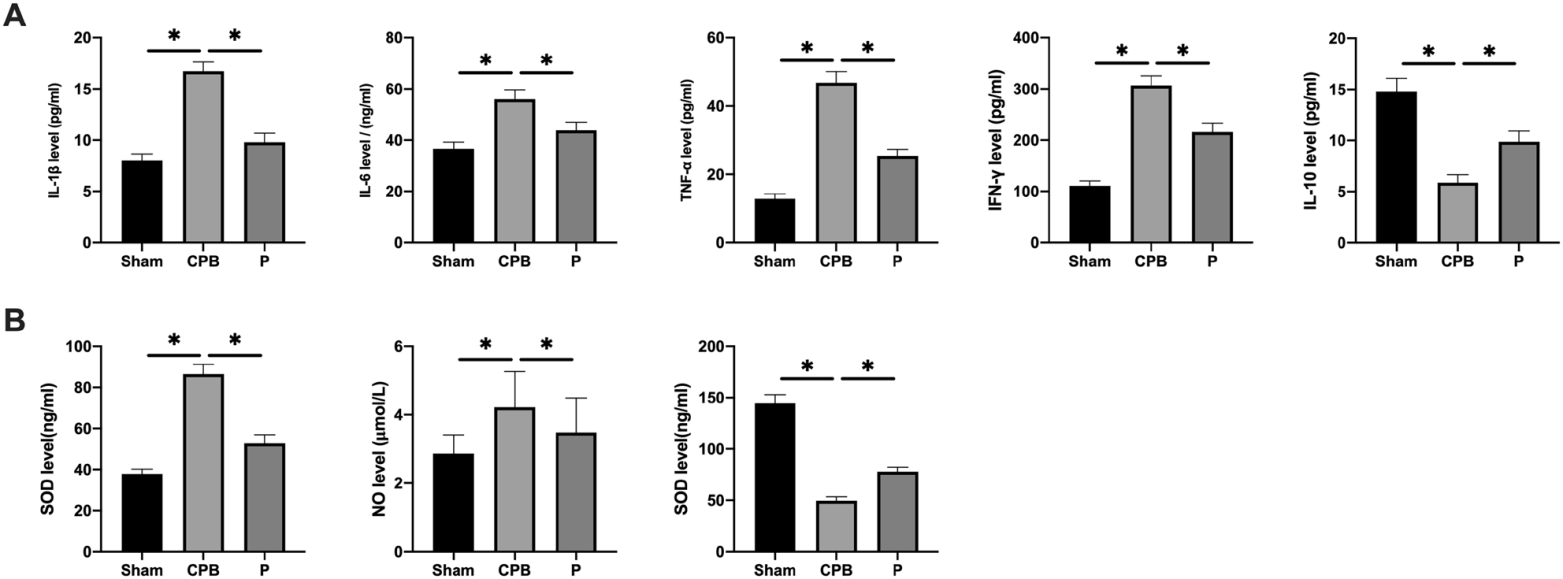
Probiotics decrease the inflammation and oxidative stress in CPB rats. (**A**) The levels of IL-1β, IL-6, TNF-α, and IFN-γ; (**B**) the relative expression level of SOD, NO and MDA. *p < 0.05.

ELISA was used to monitor the levels of inflammatory elements (Fig. 4A) and oxidative stress factors (Fig. 4B) in the rat serum. CPB surgery had increased serum concentrations of IL-1β, IL-6, TNF-α, and IFN-γ, and decreased serum concentration of IL-10 (P < 0.05), indicating severe inflammation. Furthermore, the CPB group exhibited decreased superoxide dismutase (SOD) and nitric oxide (NO) and elevated malondialdeyde (MDA) level (P < 0.05), indicating severe oxidative stress. In contrast, the P group had significantly decreased levels of IL-1β, IL-6, TNF-α, and IFN-γ, and significantly increased level of IL-10 (P < 0.05), as well as significantly promoted SOD and NO and remarkably decreased concentration of MDA (P < 0.05), indicating that probiotics reverse inflammation and oxidative stress triggered by CPB.

### Probiotics improve the neuronal apoptosis in CPB rats

**Figure 5:**
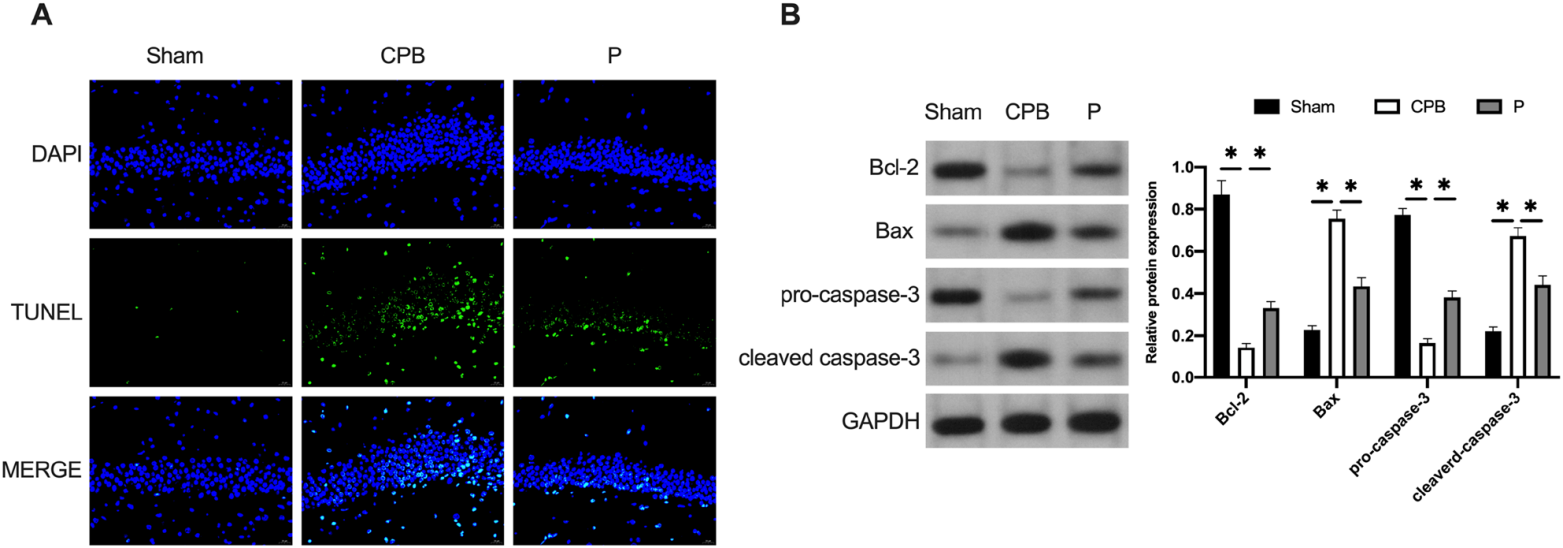
Probiotics improve neuronal apoptosis. (**A**) TUNEL staining results of rats’ brain tissue. (**B**) Western blot results of apoptosis-related proteins. *p < 0.05.

TUNEL staining was employed to detect neuronal apoptosis in brain tissue (Fig. 5A). The C group exhibited a notably higher count of positive cells compared to the S group (P < 0.05), while the P group demonstrated a significantly lower count of positive cells than the CPB group in the hippocampal brain tissue (P < 0.05). The expression of apoptosis-related factors Bcl-2, Bax, pro-caspase-3, and cleaved caspase-3 was assessed through Western blotting (Fig. 5B). The CPB group displayed a significant reduction in Bcl-2 expression and a substantial increase in Bax expression compared to the Sham group (P < 0.05). Conversely, the P group manifested a significant elevation in Bcl-2 expression and a notable reduction in Bax expression relative to the CPB group (P < 0.05). Moreover, pro-caspase-3 expression markedly decreased, and cleaved caspase-3 expression significantly increased in the CPB group compared to the Sham group (P < 0.05). In the P group, pro-caspase-3 expression significantly increased, and cleaved caspase-3 expression significantly decreased compared to the CPB group (P < 0.05). These findings indicate that probiotics have the potential to forestall neuronal degeneration and diminish neuronal apoptosis in CPB rats.

### Probiotics regulates the kynurenine metabolic proteins

**Figure 6:**
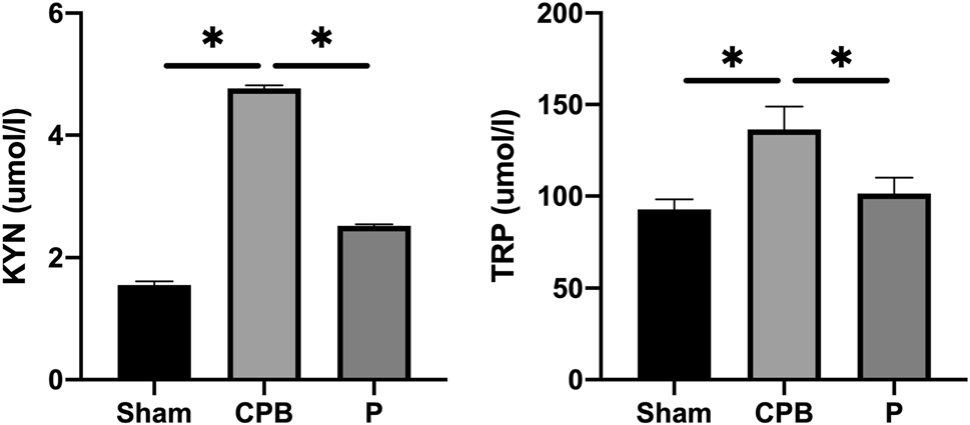
Probiotics regulates kynurenine metabolic proteins in CPB rats. HPLC/MS results of Kyn and Trp levels.

Many research investigations have highlighted anti-inflammatory role of the kynurenine metabolic pathway-signaling pathway, illustrating its capacity to inhibit apoptosis in neurons undergoing cerebral ischemia. In the context of treating POCD in rats subjected to CPB with probiotics, HPLC/MS analysis revealed a significant elevation in serum levels in the CPB group compared to those in the Sham group (Fig. 6, P < 0.05). Specifically, Kyn level was increased, while the Trp level was decreased significantly in the CPB group; intriguingly, there was a noteworthy decrease in Kyn and an increase in Trp levels ((Fig. 6, P < 0.05). Thus, probiotics may mitigate POCD in CPB rats by modulating the kynurenine metabolic pathway-signaling pathway.

## Discussion

Cognitive dysfunction is a common problem of CNS, characterized by symptoms such as confusion, anxiety, personality changes, and impaired memory^30^. POCD, which occurs in several days to weeks after CPB, was shown in decreased memory, attention, and information processing ability. POCD may cause by combined action of multiple inflammatory factors^31,32^. However, there are no reliable treatment can effectively prevent or manage POCD. Researches have shown that the inflammatory response plays a critical role in cognitive impairment in patients after surgery^33,34^. It has been reported that inhibiting the inflammatory response can effectively improve patients’ cognitive impairment^35^. However, specific drugs or biological agents that regulate the cognitive behavior of patients by modulating the inflammatory response remain to be developed.

Probiotics, when provided in sufficient quantities, are living microorganisms that contribute to the well-being of the host^36^. Probiotics performed the regulation of gut microbiota, improvement of intestinal barrier function, and modulation of immune response^37^. Specifically, probiotics have been shown to have a positive impact on inflammation, which is an important contributor to various neurological disorders^38^. Probiotics have the potential to diminish the generation of pro-inflammatory cytokines and hinder the initiation of inflammatory pathways, leading to a decrease in inflammation and an improvement in related symptoms^39^. Additionally, studies have shown that probiotics may have a beneficial effect on neurological disorders such as depression, anxiety, and cognitive impairment, potentially through their anti-inflammatory effects and regulation of the gut-brain axis^39–41^. Oral administration of specific strain of Bifidobacterium, Streptococcus thermophilus, and Lactobacillus has been proved that can effectively reduce postoperative infection rates, postoperative complication, and hospital stay duration. some experiments show that treatment with a specific strain of *Bifidobacterium* or *Lactobacillus* have a positive impact on cognitive processes and emotional behavior^42^. Bifidobacterium and Lactobacillus intake can reduce memory impairment by regulating central GABA receptor, increasing the expression of brain-derived neurotrophic factor^43,44^. Bacteroides and the closely related genus Prevotella have been shown to be associated with cognitive impairment. Probiotic capsule (BIFICO, Sine Pharmaceuticals, Shanghai, China) contains *bifidobacterium longum* (> 10^7^ colony-forming units [CFU]/210 mg),* Lactobacillus acidophil* (> 10^7^ CFU/210 mg), and Enterococcus faecalis (> 10^7^ CFU/210 mg), which have been shown to reduce postoperative cognitive impairment in elderly patients with hip or knee replacement^45^.

Overall, the use of probiotics holds great promise for improving both the prevention and treatment of various neurological disorders. Our research demonstrated that the administration of probiotics led to a significant improvement in cognitive dysfunction in CPB rats. The results of S-100β and NSE examination revealed that probiotics reduced the brain damage in CPB rats. Additionally, we demonstrated that probiotics significantly decrease inflammatory response and oxidative stress based on ELISA detection. TUNEL staining and western blotting analysis showed that probiotics attenuated the hippocampal neuronal apoptosis. These findings suggested that probiotics could improve the POCD-related cognitive dysfunction in CPB rats.

KYN is a metabolite of the essential amino acid TRP in the human body. The KP is important in TRP metabolism of brain. This pathway is composed of different metabolites and various enzymes and is regulated by pro-inflammatory mediators and hormones, which activate or change the synthesis of metabolites in the pathway and regulate neurotransmitter release and functional execution. Moreover, many studies have found that the physicochemical changes in various CNS diseases are related to KP pathway metabolic disorders^46–48^. Therefore, the KP pathway plays a critical role in cognitive function.

SIRS is a significant pathological and physiological change after CPB heart surgery, and it is also an important factor in the damage to multiple target organs in the body^49^. Inflammatory elements encompassing TNF-α, IL-1β and IL-6 are related with SIRS response. These inflammatory factors can destroy the blood brain barrier (BBB), leading to brain tissue damage^50^. Numerous inflammatory factors infiltrated the brain tissue via diverse pathways, triggering the activation of glial cells and initiating a cascade of immune responses that lead to an overproduction of inflammatory mediators within CNS, and lead to neuronal damage, resulting in a decline in behavioral and memory abilities^51^. During CPB, because blood is exposed to the external environment, the body's immune system is activated, leading to SIRS during the perioperative period, and many pro-inflammatory factors such as TNF-α and IL-1β are released. In the blood after CPB surgery, endotoxins cause the release of cytokines. These cause an increase in the quinolinic acid (QUIN)/KYNA ratio. QUIN is an N-methyl-D-aspartate (NMDA) receptor activator, and injection of it can cause a decline in cognitive function accompanied by neuronal loss, similar with cognitive impairment. KYNA is the only endogenous inhibitor of NMDA receptors and plays an important role in the regulation of neural function. KYNA can block the toxicity of excitatory amino acids by inhibiting NMDA receptors, thereby preventing damage to brain tissues and protecting the nervous system^52^. Zwilling et al.^53^found that an increase in KYNA concentration and a decrease in QUIN concentration in the brain can significantly reduce the occurrence of Alzheimer disease (AD), Huntington’s disease, and neurodegeneration. Solvang et al.^54^found that the KYN/TRP ratio (KTR) and folic acid levels were positively correlated with overall cognitive impairment in the elderly. Forrest et al.^55^found that the KTR in peripheral blood undergoing CPB increased significantly at 18 h and 6 days after CPB, indicating an increase in IDO activity; KYNA and 3-HAA increased significantly on the 6th day, and 3-HAA increased more significantly; Folate and TNF-α also increased on the 6th day post-CPB. Their regression analysis showed that the substances in the KP pathway were important predictors of cognitive function changes after surgery. Forrest proved that the KP pathway and folate are involved in cognitive impairment after heart surgery, and more importantly, inhibition of the KP pathway can prevent brain damage caused by ischemia and reduce central inflammation-related mortality, suggesting that the KP pathway can be used as an important potential target for the promotion of drugs for the prevention and treatment of brain damage or POCD. In our study, we found that probiotics could regulate the levels of KYN and TRP in Rats serum, balance the ratio of KYN/TRP, leading to reduce the inflammatory factors and promote the cognitive impairment.

To summarize, our study suggests that probiotics have neuroprotective effects against CPB-induced POCD brain damage, partially through modulation of the kynurenine metabolic pathway. Our research performed novel finding for molecular mechanisms and signaling pathways underlying the beneficial effects of probiotics in POCD brain damage and may offer a novel therapeutic target. Future studies should investigate other potential relationships between the gut microbiota and the kynurenine metabolic pathway in improving the specific mechanisms of POCD.

## Data Availability

The datasets of our study are available from the corresponding author on reasonable request.

## Abbreviations

POCD: Postoperative cognitive dysfunction
ELISA: Enzyme-linked immunosorbent assays
TUNEL: TdT-mediated dUTP nick end-labeling
HPLC/MS: High-performance liquid chromatography/mass spectrometry
H&E: Hematoxylin and eosin
NSE: Neuron-specific enolase
CPB: Cardiopulmonary bypass
PND: Perioperative neurocognitive disorders
POD: Postoperative delirium
NCD: Neurocognitive disorders
TRP: Tryptophan
TDO: Tryptophan-2,3-dioxygenase
IDO: Indoleamine-2,3-dioxygenase
KYNA: Kynurenic acid
QUIN: Quinolinic acid
MAP: Mean arterial pressure
CVP: Central venous pressure
IL-1β: Interleukin
TNF-α: Tumor necrosis factor alpha
IFN-γ: Interferon
S-100β: Protein-100β
PaCO_2_: Pressure of carbon dioxide
PaO_2_: Partial pressure of oxygen
KYN: Kynurenine
SOD: Superoxide dismutase
NO: Nitric oxide
MDA: Malondialdeyde
NMDA: *N*-methyl-d-aspartate
WHO: World Health Organization
BBB: Blood brain barrier

## References

[1] Baker RA, Nikolic A, Onorati F (2020). 2019 EACTS/EACTA/EBCP guidelines on cardiopulmonary bypass in adult cardiac surgery: A tool to better clinical practice. Eur. J. Cardiothorac. Surg..

[2] Gaudino M, Rahouma M, Di Mauro M (2019). early versus delayed stroke after cardiac surgery: A systematic review and meta-analysis. J. Am. Heart Assoc..

[3] Pataraia E, Jung R, Aull-Watschinger S (2018). Seizures after adult cardiac surgery and interventional cardiac procedures. J. Cardiothorac. Vasc. Anesth..

[4] Rudolph JL, Jones RN, Levkoff SE (2009). Derivation and validation of a preoperative prediction rule for delirium after cardiac surgery. Circulation.

[5] DeVita MA, Robinson LR, Rehder J (1993). Incidence and natural history of phrenic neuropathy occurring during open heart surgery. Chest.

[6] McKhann GM, Grega MA, Borowicz LM (2005). Is there cognitive decline 1 year after CABG? Comparison with surgical and nonsurgical controls. Neurology.

[7] Evered L, Silbert B, Knopman DS (2018). Recommendations for the nomenclature of cognitive change associated with anaesthesia and surgery-2018. Br. J. Anaesth..

[8] Needham MJ, Webb CE, Bryden DC (2017). Postoperative cognitive dysfunction and dementia: What we need to know and do. Br. J. Anaesth..

[9] Subramaniyan S, Terrando N (2019). Neuroinflammation and perioperative neurocognitive disorders. Anesth. Analg..

[10] Rasmussen LS (2006). Postoperative cognitive dysfunction: Incidence and prevention. Best Pract. Res. Clin. Anaesthesiol..

[11] Giacinto O, Satriano U, Nenna A (2019). Inflammatory response and endothelial dysfunction following cardiopulmonary bypass: Pathophysiology and pharmacological targets. Recent Pat. Inflamm. Allergy Drug Discov..

[12] Salameh A, Dhein S, Dähnert I (1945). Neuroprotective strategies during cardiac surgery with cardiopulmonary bypass. Int. J. Mol. Sci..

[13] Merino JG, Latour LL, Tso A (2013). Blood-brain barrier disruption after cardiac surgery. AJNR Am. J. Neuroradiol..

[14] Berger M, Terrando N, Smith SK (2018). Neurocognitive function after cardiac surgery: From phenotypes to mechanisms. Anesthesiology.

[15] Abrahamov D, Levran O, Naparstek S (2017). Blood-brain barrier disruption after cardiopulmonary bypass: Diagnosis and correlation to cognition. Ann. Thorac. Surg..

[16] Camilleri M (2021). Human intestinal barrier: Effects of stressors, diet, prebiotics, and probiotics. Clin. Transl. Gastroenterol..

[17] Mao L, Zeng Q, Su W (2021). Elevation of miR-146a inhibits BTG2/BAX expression to ameliorate postoperative cognitive dysfunction following probiotics (VSL#3) treatment. Mol. Neurobiol..

[18] Wang P, Yin X, Chen G (2021). Perioperative probiotic treatment decreased the incidence of postoperative cognitive impairment in elderly patients following non-cardiac surgery: A randomised double-blind and placebo-controlled trial. Clin. Nutr..

[19] Zhang X, Chen Y, Tang Y (2023). Efficiency of probiotics in elderly patients undergoing orthopedic surgery for postoperative cognitive dysfunction: A study protocol for a multicenter, randomized controlled trial. Trials.

[20] Liu L, Shang L, Jin D (2022). General anesthesia bullies the gut: A toxic relationship with dysbiosis and cognitive dysfunction. Psychopharmacology.

[21] Heimberger AB, Lukas RV (2023). The kynurenine pathway implicated in patient delirium: possible indications for indoleamine 2,3 dioxygenase inhibitors. J. Clin. Invest..

[22] Wu W, Nicolazzo JA, Wen L (2013). Expression of tryptophan 2,3-dioxygenase and production of kynurenine pathway metabolites in triple transgenic mice and human Alzheimer’s disease brain. PLoS One.

[23] Schwarcz R, Guidetti P, Sathyasaikumar KV (2010). Of mice, rats and men: Revisiting the quinolinic acid hypothesis of Huntington’s disease. Prog. Neurobiol..

[24] Öztürk M, Yalın Sapmaz Ş, Kandemir H (2021). The role of the kynurenine pathway and quinolinic acid in adolescent major depressive disorder. Int. J. Clin. Pract..

[25] Ferreira FS, Schmitz F, Marques EP (2020). Intrastriatal quinolinic acid administration impairs redox homeostasis and induces inflammatory changes: Prevention by kynurenic acid. Neurotox. Res..

[26] Sun YJ, Cao HJ, Song DD (2013). Probiotics can alleviate cardiopulmonary bypass-induced intestinal mucosa damage in rats. Dig. Dis. Sci..

[27] Li X, Sun Y, Jin Q (2019). Kappa opioid receptor agonists improve postoperative cognitive dysfunction in rats via the JAK2/STAT3 signaling pathway. Int. J. Mol. Med..

[28] Garcia JH, Wagner S, Liu KF (1995). Neurological deficit and extent of neuronal necrosis attributable to middle cerebral artery occlusion in rats: Statistical validation. Stroke.

[29] Bederson JB, Pitts LH, Tsuji M (1986). Rat middle cerebral artery occlusion: Evaluation of the model and development of a neurologic examination. Stroke.

[30] Lin Y, Chen M, Peng Y (2021). Feeding intolerance and risk of poor outcome in patients undergoing cardiopulmonary bypass surgery. Br. J. Nutr..

[31] Qin J, Ma Q, Ma D (2020). Low-dose sevoflurane attenuates cardiopulmonary bypass (CPB)-induced postoperative cognitive dysfunction (POCD) by regulating hippocampus apoptosis via PI3K/AKT pathway. Curr. Neurovasc. Res..

[32] Evered LA, Silbert BS (2018). Postoperative cognitive dysfunction and noncardiac surgery. Anesth. Analg..

[33] Wang Z, Meng S, Cao L (2018). Critical role of NLRP3-caspase-1 pathway in age-dependent isoflurane-induced microglial inflammatory response and cognitive impairment. J. Neuroinflamm..

[34] Feng X, Valdearcos M, Uchida Y (2017). Microglia mediate postoperative hippocampal inflammation and cognitive decline in mice. JCI Insight.

[35] Wang XF, Lin Q, Wang GH (2022). Electroacupuncture stimulation suppresses postoperative inflammatory response and hippocampal neuronal injury. Mediators Inflamm..

[36] Williams NT (2010). Probiotics. Am. J. Health Syst. Pharm..

[37] Quigley EMM (2019). Prebiotics and probiotics in digestive health. Clin. Gastroenterol. Hepatol..

[38] Zhou J, Li M, Chen Q (2022). Programmable probiotics modulate inflammation and gut microbiota for inflammatory bowel disease treatment after effective oral delivery. Nat. Commun..

[39] Den H, Dong X, Chen M (2020). Efficacy of probiotics on cognition, and biomarkers of inflammation and oxidative stress in adults with Alzheimer’s disease or mild cognitive impairment—A meta-analysis of randomized controlled trials. Aging.

[40] Cheng LH, Liu YW, Wu CC (2019). Psychobiotics in mental health, neurodegenerative and neurodevelopmental disorders. J. Food Drug Anal..

[41] Shahbazi R, Yasavoli-Sharahi H, Alsadi N (2020). Probiotics in treatment of viral respiratory infections and neuroinflammatory disorders. Molecules.

[42] Savignac HM, Tramullas M, Kiely B, Dinan TG, Cryan JF (2015). Bifidobacteria modulate cognitive processes in an anxious mouse strain. Behav. Brain Res..

[43] Savignac HM, Corona G, Mills H (2013). Prebiotic feeding elevates central brain derived neurotrophic factor, N-methyl-D-aspartate receptor subunits and D-serine. Neurochem. Int..

[44] Bravo JA, Forsythe P, Chew MV (2011). Ingestion of *Lactobacillus* strain regulates emotional behavior and central GABA receptor expression in a mouse via the vagus nerve. Proc. Natl. Acad. Sci. USA..

[45] Hu L, Luo M, Huang H (2023). Perioperative probiotics attenuates postoperative cognitive dysfunction in elderly patients undergoing hip or knee arthroplasty: A randomized, double-blind, and placebo-controlled trial. Front. Aging Neurosci..

[46] Venkatesan D, Iyer M, Narayanasamy A (2020). Kynurenine pathway in Parkinson’s disease—An update. eNeurologicalSci.

[47] Fuertig R, Ceci A, Camus SM (2016). LC-MS/MS-based quantification of kynurenine metabolites, tryptophan, monoamines and neopterin in plasma, cerebrospinal fluid and brain. Bioanalysis..

[48] Almulla AF, Vasupanrajit A, Tunvirachaisakul C (2022). The tryptophan catabolite or kynurenine pathway in schizophrenia: Meta-analysis reveals dissociations between central, serum, and plasma compartments. Mol. Psychiatr..

[49] Zeng ZH, Yu XY, Liu XC (2022). Effect of CPB glucose levels on inflammatory response after pediatric cardiac surgery. BMC Cardiovasc. Disord..

[50] Sistino JJ, Acsell JR (1999). Systemic inflammatory response syndrome (SIRS) following emergency cardiopulmonary bypass: A case report and literature review. J. Extra Corpor. Technol..

[51] Wang W, Zhang XY, Feng ZG (2017). Overexpression of phosphodiesterase-4 subtypes involved in surgery-induced neuroinflammation and cognitive dysfunction in mice. Brain Res. Bull..

[52] Yi SQ, Yang M, Duan KM (2015). Immune-mediated metabolic kynurenine pathways are involved in the postoperative cognitive dysfunction after cardiopulmonary bypass. Thorac. Cardiovasc. Surg..

[53] Zwilling D, Huang SY, Sathyasaikumar KV (2011). Kynurenine 3-monooxygenase inhibition in blood ameliorates neurodegeneration. Cell.

[54] Solvang SH, Nordrehaug JE, Tell GS (2019). The kynurenine pathway and cognitive performance in community-dwelling older adults. The Hordaland Health Study. Brain Behav. Immun..

[55] Forrest CM, Mackay GM, Oxford L (2011). Kynurenine metabolism predicts cognitive function in patients following cardiac bypass and thoracic surgery. J. Neurochem..

